# Hierarchy‐Assembled Dual Probiotics System Ameliorates Cholestatic Drug‐Induced Liver Injury via Gut‐Liver Axis Modulation

**DOI:** 10.1002/advs.202200986

**Published:** 2022-04-17

**Authors:** Qi‐Wen Chen, Qian‐Ru Li, Meng‐Wei Cao, Jian‐Hua Yan, Xian‐Zheng Zhang

**Affiliations:** ^1^ Key Laboratory of Biomedical Polymers of Ministry of Education and Department of Chemistry Wuhan University Wuhan 430072 P. R. China

**Keywords:** cholestasis, intestinal tract, liver injury, polymer microsphere, probiotics

## Abstract

Cholestatic drug‐induced liver injury (DILI) induced by drugs or other xenobiotics is a severe and even fatal clinical syndrome. Here, living materials of hierarchy‐assembled dual probiotics system are fabricated by sequentially encapsulating probiotic *Lactobacillus delbrueckii* subsp. *bulgaricus* (LDB) and *Lactobacillus rhamnosus* GG (LGG) into Ca^2+^‐complexed polymer microspheres for effective prevention of cholestatic DILI. Upon entering intestinal tract of the constructed living materials, LGG is released because of pH‐triggered dissolution of outer enteric polymer coating. The released LGG can inhibit hepatic bile acids (BAs) synthesis by activating intestinal farnesoid X receptor‐fibroblast growth factor 15（FGF‐15) signaling pathway. BAs excretion is also facilitated by LGG through increasing the abundance of bile salt hydrolase (BSH)‐active gut commensal bacteria. Furthermore, exposed positively‐charged chitosan shell can absorb the excessive BAs via electrostatic interaction, which leads to steady BAs fixation by the imprisoned LDB, decreasing the total BAs amounts in enterohepatic circulation. Together, the fabricated living materials, obtained here, can effectively prevent cholestatic DILI through dredging cholestasis via gut‐liver axis modulation. The therapeutic effect is demonstrated in *α*‐naphthylisothiocyanate and clinical antiepileptic drug valproate acid‐induced cholestatic DILI mouse models, which reveal the great potential for effective cholestatic DILI management.

## Graphical abstract

Hierarchy‐assembled dual probiotics system is developed, which reveal great potential to ameliorate cholestatic drug‐induced liver injury via gut‐liver axis modulation.

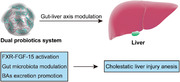

## 1 Introduction

Cholestatic drug‐induced liver injury (DILI) triggered by drugs or other xenobiotics is a common clinical challenge with respect to diagnosis and treatment.^[^ ^1^, ^2^ ^]^ According to the liver injury phenotypes and presumed mechanisms of action of administrated chemical compounds, the drug hepatotoxicity can be typically classified as two categories, including the predictable event featuring with acute liver failure caused by acetaminophen, and the idiosyncratic event which is unpredictable.^[^ ^3^ ^]^ Currently, the commonly used intervention strategies are to suppress the hepatic inflammatory effect by using anti‐inflammatory drugs or nanomaterials, or to suspend the offending drugs and avoid re‐exposure.^[^ ^4^ ^]^ In fact, these therapeutic methods are only palliatives and usually restricted. Especially, no definitive therapy is available in the idiosyncratic DILI. Therefore, developing new therapeutic strategies for idiosyncratic DILI intervention is urgently needed. It was reported that the perturbation of bile acids (BAs) homeostasis is a common early event in DILI.^[^ ^5^ ^]^ Particularly, drug‐induced cholestasis is one of the prominent characteristic and risk factor in the idiosyncratic DILI.^[^ ^2^, ^6^ ^]^ Beyond causing serious liver injury, the long period of cholestasis in liver tissue is very possible to finally result in more serious liver diseases like choleplania, primary sclerosing cholangitis (PSC), hepatic fibrosis, cirrhosis, and cancers (including biliary duct cancer, hepatic cancer, and pancreatic cancer), et al.^[^ ^7^ ^]^ In view of these, desludging the hepatic and intestinal BAs drainage could be a promising therapeutic opportunity in the prevention of cholestatic DILI.^[^ ^8^ ^]^

Primary BAs are synthesized in hepatic tissue from cholesterol and conjugated with taurine or glycine to generate the conjugated BAs. These molecules can be released into the intestinal tract to aid in the absorption of lipids and vitamins. Then, over 95% BAs are reabsorbed by ileum and transported to the liver via the enterohepatic circulation.^[^ ^9^ ^]^ The remaining primary BAs are enzymatically deconjugated and modified by the resided gut bacteria to produce a group of secondary BAs, which in turn regulate the hepatic BAs metabolism and affect multiple enterohepatic diseases.^[^ ^10^, ^11^ ^]^ As a matter of course, a promising therapeutic opportunity could be provided by modulating biliary gut‐liver axis circulation and gut microbiota mediated BAs metabolism for effective intervention of cholestasis‐related diseases. For example, in order to dispose the cholestasis, commercial positively charged resin polymers like cholestyramine and colestipol are mostly applied to scavenge BAs through electrostatically adsorbing the free BAs in intestinal tract.^[^ ^12^ ^]^ In fact, many negatively charged molecules like proteins and fatty acids can disturb the polymeric sequestration of BAs, so that the clearance effect is significantly limited. To achieve the ideal intervention result, a high dose of these resin polymer drug is necessary to be administrated, which inevitably interferes with normal nutrient absorption. Additionally, these polymeric drugs only take away the intestinal BAs, while the hepatic BAs are rarely stirred because the cholesterol metabolism could be activated and replenish the BAs pool.^[^ ^13^ ^]^ Natural ursodeoxycholic acid (UDCA) is the most established medical treatment for cholestasis anesis by promoting hepatic BAs excretion and decreasing intestinal BAs reabsorption,^[^ ^14^ ^]^ but the independent UDCA therapy could be also restricted by inadequate response and drug resistance.^[^ ^15^ ^]^

Recently, by uniting the alive traits of microorganisms with the versatile properties of functional materials, the customized materials‐assisted microorganisms (MAMO) were well fabricated,^[^ ^16^ ^]^ and emergingly applied in biosensing,^[^ ^17^ ^]^ self‐repairing and regeneration,^[^ ^18^ ^]^ protein production,^[^ ^19^ ^]^ antipathogens, and diseases treatment.^[^ ^20^ ^]^ Particularly, by utilizing the unique ability of microorganisms, many intractable diseases have the chance to be well treated, including kidney failure,^[^ ^21^ ^]^ colon cancer,^[^ ^22^ ^]^ colitis,^[^ ^23^ ^]^ and alcoholic liver disease,^[^ ^24^ ^]^ etc. These works indicated the feasibility of employing microorganisms and functional materials, especially life unit‐involved polymers (LIP), to rationally build living materials to treat DILI through relieving cholestasis. Previous literatures reported that some probiotics like *Lactobacillus delbrueckii* subsp. *bulgaricus* (LDB) can actively gather the BAs by multiple mechanisms including bacterial transmembrane proton gradient driven transportation and bacterial S‐layer proteins mediated binding.^[^ ^25^ ^]^ Additionally, it was also found that *Lactobacillus rhamnosus* GG (LGG) can inhibit hepatic BAs synthesis and enhance BAs secretion through activating intestinal farnesoid X receptor (FXR)‐fibroblast growth factor 15 (FGF‐15) signaling pathway and regulating gut microbiota‐mediated primary BAs deconjugation.^[^ ^11^, ^26^ ^]^ Therefore, combining LDB and LGG has the potential to thoroughly dredge the blockage of BAs in liver and intestine via modulating BAs circulation in gut‐liver axis and facilitating gut microbiota‐guided BAs metabolism.

**Scheme 1:**
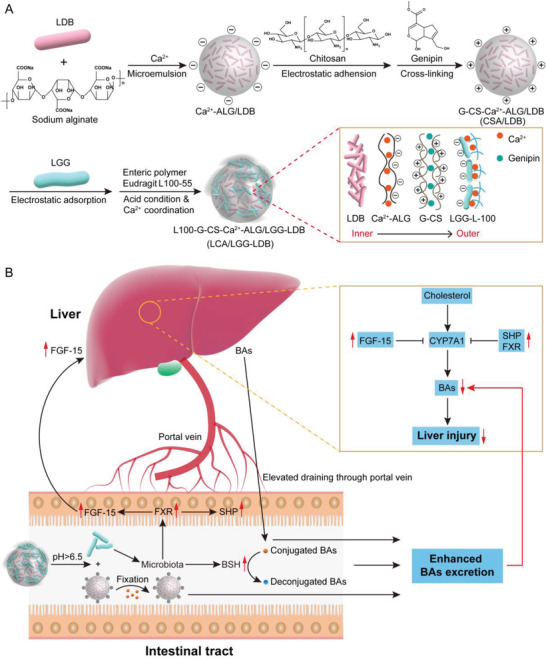
Schematic illustration of the synthesis procedure of dual probiotics‐incorporated living materials and the therapeutic mechanism on cholestatic DILI. A) The fabrication process of probiotics‐based living materials through hierarchically assembling LDB and LGG into Ca^2+^‐coordinated polymer microspheres. B) Gut‐liver axis modulation mediated hepatic BAs reduction by the synthesized living materials for alleviation of cholestatic DILI.

In this study, living materials of hierarchy‐assembled dual probiotics system were constructed by orderly encapsulating LDB and LGG into Ca^2+^‐coordinated polymer microspheres (**Scheme** **1**A). LDB was incorporated into calcium alginate microspheres via microemulsion method. Then, the positively charged chitosan was modified onto calcium alginate microspheres surface by electrostatic interaction, which was further immobilized via genipin‐mediated crosslinking. The surface charges of calcium alginate microspheres were reversed and used for absorbing negatively charged LGG and commercial enteric polymers of Eudragit L100‐55. In order to stably incorporate LGG, Ca^2+^ was introduced to chelate enteric polymers to form outer coating for LGG encapsulation and protection. Upon entering intestinal tract of the fabricated living materials (designated as LCA/LGG‐LDB), LGG was released due to the pH‐dependent dissolution of enteric polymer coating. The released LGG can decrease hepatic BAs synthesis through activating intestinal FXR‐FGF‐15 signaling pathway, and promote BAs excretion through enhancing the richness of bile salt hydrolase (BSH)‐active gut commensal microbes. The followingly exposed chitosan shell can prolong the retention time of calcium alginate microspheres in intestinal tract, play crucial role to absorb the excessive BAs, promote the steady fixation of BAs by the imprisoned LDB in calcium alginate microspheres, and finally decrease the total BAs amounts in enterohepatic circulation along with the excretion out of LDB‐trapped calcium alginate microspheres (Scheme 1B). The effective cholestatic DILI alleviation was investigated in *α*‐naphthylisothiocyanate (ANIT) and clinical antiepileptic drugs valproate acid (VPA)‐induced mouse models of cholestatic DILI by employing LCA/LGG‐LDB. It was found that LCA/LGG‐LDB can significantly dredge cholestasis via gut‐liver axis modulation, indicating great potential to serve as general strategy for cholestatic DILI treatment.

## 2 Results and Discussion

### 2.1 Synthesis and Characterization of Hierarchy‐Assembled Dual Probiotics System

**Figure 1:**
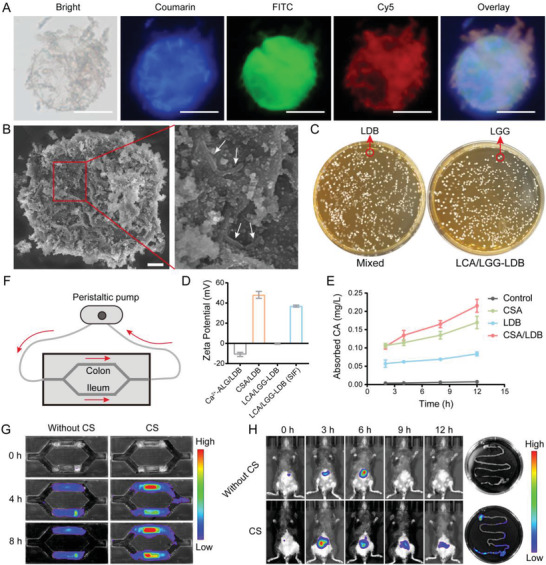
Synthesis of LCA/LGG‐LDB and corresponding properties characterization. A) Fluorescent colocalization of coumarin‐labeled LDB, FITC‐labeled chitosan, and Cy5‐labeled LGG in dual probiotics system (Scale bar: 50 µm). B) SEM image of LCA/LGG‐LDB and corresponding region zoom (Scale bar: 5 µm). C) The bacterial colonies of mixed pure LDB and LGG, as well as LCA/LGG‐LDB (10^5^ times dilution). D) Zeta potential of different materials during fabrication of LCA/LGG‐LDB. E) Absorption ability of CSA, LDB, and CSA/LDB toward model BAs of CA. F,G) The retention experiments of LCA/LGG‐LDB with or without chitosan (CS) modification in ileum and colon‐sticked grooves using a microfluids device (Performed in SIF solution, LDB was labeled with Cy5.5). H) In vivo intestinal retention experiments of LCA/LGG‐LDB with or without CS modification (LDB was labeled with Cy5.5).

In order to maintain bacterial bioactivity in harsh gastrointestinal environment and meanwhile conduct required substances exchange between bacteria and surrounding environment, during the fabrication of dual probiotic system, LDB was first encapsulated into Ca^2+^‐complexed alginate microspheres via microemulsion method. As shown in Figure S1A (Supporting Information), coumarin‐labeled LDB bacteria (blue) were encapsulated into calcium alginate microspheres with diameter over 50 µm and without leaving free bacteria, proving that LDB bacteria were completely encapsulated. Next, chitosan was decorated onto the surface of LDB‐contained calcium alginate microspheres via electrostatic interaction. Genipin was used to crosslink chitosan for stabilizing the modified microspheres. Fluorescence image showed that fluorescein isothiocyanate (FITC)‐labeled chitosan (green) was evenly coated onto calcium alginate microspheres surface, demonstrating the successful modification of chitosan (Figure S1B, Supporting Information). The modification of chitosan can convert the negatively charged surface of calcium alginate microspheres to the positively charged surface. The charges reversal is beneficial to incorporate negatively‐charged LGG and commercial enteric polymers of Eudragit L100‐55 that were used to protect LGG in gastric fluid and appropriately release LGG in alkalescent intestinal tract. Enteric polymers were further coordinated by Ca^2+^ to lock LGG onto the surface of microspheres tightly. Fluorescence image displayed that cyanine 5 (Cy5)‐labeled LGG bacteria (red) were incorporated onto the surface of chitosan‐modified calcium alginate microspheres by enteric polymers (Figure S1C, Supporting Information). The fluorescent colocalization of coumarin‐labeled LDB, FITC‐labeled chitosan and Cy5‐labeled LGG finally proved that LCA/LGG‐LDB was successfully synthesized (**Figure** **1**A; and Figure S1C, Supporting Information). Considering the possible antibacterial ability of the used components, bacterial cytotoxicity of various components was evaluated (Figure S2, Supporting Information). The results demonstrated that the growth of both LGG and LDB were negligibly affected by chitosan and sodium alginate. Enteric polymers also showed no antibacterial effect on LGG growth, demonstrating that the incorporation process of LGG by enteric polymers will not affect bacterial activity. Scanning electron microscope (SEM) image observed the spherical morphology of LCA/LGG‐LDB, which contained uninjured bacteria (Figure 1B). The bacterial activity was further evaluated by coating LCA/LGG‐LDB onto Man Rogosa Sharpe (MRS) agar plates. Both LGG and LDB trapped in LCA/LGG‐LDB maintained the consistent colonies comparing to the treatment of physical mixture of two bacteria, demonstrated that no bacterial activity damage was caused by the hierarchical encapsulation process (Figure 1C). Additionally, the outer LGG activity in different physiological conditions were further assessed. Only LGG was encapsulated into the fabricated living materials (Abbreviated as LCA/LGG) for conveniently counting. As shown in Figure S3 (Supporting Information), by the dilution coating method in MRS agar plates, the slightly decreased LGG colonies were displayed in stimulated intestinal fluid (SIF), stimulated BAs fluid (BA), and stimulated gastric fluid (SGF), testifying that LGG activity was markedly protected by outer coating of Ca^2+^‐coordinated enteric polymers. Above results fully characterized that the biocompatible living materials of LCA/LGG‐LDB was successfully constructed, and the bacterial activity can be effectively sustained by Ca^2+^‐coordinated polymers.

According to construction principle, electrostatic interaction is the main assembling force of LCA/LGG‐LDB. The zeta potential was tested by orderly mixing the used components for expediently monitoring the potential changes during establishment of dual probiotics system (Figure 1D). The zeta potential of the used components was shown in Figure S4 (Supporting Information). In the mixture, the zeta potential showed the negatively charged calcium alginate (Ca^2+^‐ALG/LDB) about −10.91 mV, while the zeta potential was reversed into positively charged ≈48.05 mV after chitosan modification (G‐CS‐Ca^2+^‐ALG/LDB, CSA/LDB). When coating with enteric polymers, the zeta potential was changed into negatively charged of −0.55 mV, suggesting that the final LCA/LGG‐LDB is negatively charged. The zeta potential of LCA/LGG‐LDB was again converted to positively charged after incubating in SIF, meaning that the outer enteric polymers was dissolved. The dissolution effect of LCA/LDB‐LGG was measured in SGF and SIF. As shown in Figure S5 (Supporting Information), LCA/LGG‐LDB displayed unchanged morphology after incubating in SGF, but the outer enteric polymers were dissolved and the incorporated LGG was released after incubating in SIF. The phenomenon was ascribed to the pH‐dependent dissolution feature of enteric polymers. After dissolution of enteric polymers, the exposed positively‐charged microspheres could actively contribute to prolong intestinal retention time and promote BAs fixation. The BAs fixation effect was tested by furfural‐based colorimetry method. As shown in Figure 1E, both pure LDB and CSA can effectively absorb BAs in phosphate buffer solution (PBS), and the absorption effect was enhanced after encapsulating LDB into CSA. The intestinal retention effect was investigated by using a microfluids device sticking with ileum and colon tissues in the grooves (Figure 1F). As shown in Figure 1G, compared to LCA/LGG‐LDB without chitosan modification, the intact LCA/LGG‐LDB (Cy5.5 labeling LDB) dispersed in SIF presented increased Cy5.5 fluorescence intensity in both ileum and colon tissues, which confirmed the excellent tissue adhesion of the exposed positively‐charged microspheres. Furthermore, in vivo intestinal retention effect was evaluated by gavaging mouse with LCA/LGG‐LDB or LCA/LGG‐LDB without chitosan modification. The results of In Vivo Imaging System (IVIS) showed the delayed Cy5.5 fluorescence disappearance of LCA/LGG‐LDB but rapid fluorescence weakening of LCA/LGG‐LDB without chitosan modification. The retained fluorescence was also observed in excised gastrointestinal tissue in the treatment of LCA/LGG‐LDB. While in LCA/LGG‐LDB without chitosan modification, the fluorescence completely disappeared (Figure 1H). These evidences suggested that the synthesized dual probiotics system possesses the abilities to prolong retention time and scavenge excess BAs in intestinal tract. By labeling LDB with Cy5, the final fate of LDB was evaluated. The intact morphology of the synthesized microspheres was observed in excreted feces, and Cy5 fluorescence image showed that LDB was still imprisoned into the microspheres (Figure S6, Supporting Information), suggesting that LDB was finally excreted out along with feces.

### 2.2 Remission of ANIT‐Induced Acute Liver Bile Duct Injury by LCA/LGG‐LDB

**Figure 2:**
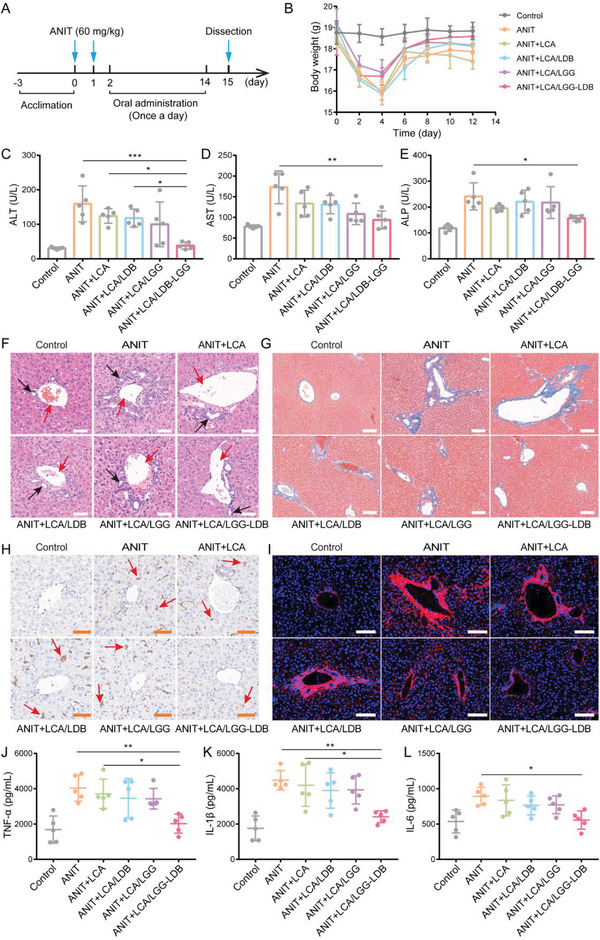
Amelioration of ANIT‐induced acute liver bile duct injury in mouse model. A) The therapeutic process of ANIT‐induced mice model of cholestasis and acute liver bile duct injury. B) The changes of mice body weight during treatment. Liver function‐related enzymes concentration of C) ALT, D) AST, and E) ALP. F) H&E staining of liver tissues in different treatment groups (Red arrows: interlobular vein (IV); Black arrows: interlobular bile duct (IB); Scale bar: 50 µm). G) Hepatic Masson staining in different treatment groups (Scale bar: 100 µm). H) Hepatic immunohistochemical staining of F4/80 marker‐positive macrophages (red arrows) in different treatment groups (Scale bar: 50 µm). I) Hepatic immunofluorescence staining of collagen‐I in different groups (Scale bar: 100 µm). The hepatic inflammatory factors of J) TNF‐*α*, K) IL‐1*β*, L) IL‐6. The data were presented as the mean ± s.d., *n* = 5. The statistical significance was calculated via one‐way ANOVA with Tukey's multiple comparisons test. *P < 0.05, **P < 0.01, ***P < 0.001.

A typical mouse model of ANIT‐induced intrahepatic cholestasis and acute liver bile duct injury was constructed to investigate the potential therapeutic effect of synthesized dual probiotic system.^[^ ^27^ ^]^ The therapeutic process was shown in **Figure** **2**A. After treating with ANIT for 48 h, the synthetic LCA/LGG‐LDB and other control group materials (including LCA/LGG, LCA/LDB, LCA, and saline (ANIT)) were administrated for 12 days. The body weight changes were recorded during treatment. As shown in Figure 2B, mice gavaged with LCA/LGG‐LDB showed rapid body weight recovery in contrast to other control groups. The liver function‐associated enzymes were tested for evaluating the liver injury. Results exhibited that the main liver function‐associated enzymes like alanine aminotransferase (ALT), aspartate aminotransferase (AST), and alkaline phosphatase (ALP) were significantly increased after ANIT treatment. The increment was weakly counteracted by the treatment of LCA, LCA/LDB, and LCA/LGG. While orally administrating with LCA/LGG‐LDB, the rising of liver function‐associated enzymes was markedly restrained (Figure 2C–E). Also, the bilirubin levels including total bilirubin (TBIL) and direct bilirubin (DBIL) were obviously elevated after ANIT treatment, which were less restored by LCA, LCA/LDB, and LCA/LGG treatments. Whereas, the enhancement of bilirubin was significantly inhibited by LCA/LGG‐LDB (Figure S7, Supporting Information). Liver histology of hematoxylin and eosin (H&E) staining showed normal tissue structures in control group, but in ANIT and other control groups (LCA, LCA/LDB, LCA/LGG), the wounded tissue structures in the Glisson's sheath and the adjacent liver parenchyma were observed, including liver cell necrosis, sinusoids dilation, portal edema, diffuse bile duct hyperplasia, and mild neutrophilic infiltration. The severe alterations of liver tissue architectures were prominently improved by cotreating with LCA/LGG‐LDB (Figure 2F). After ANIT treatment, the portal tracts also displayed marked fibrosis as observed by Masson staining (Figure 2G). The immunofluorescence staining of hepatic collagen I, the most abundant extracellular matrix protein in the fibrotic liver, also showed high expression in ANIT group, which was consistent with Masson staining results, demonstrating the severe fibrosis caused by ANIT (Figure 2I). The hepatic fibrosis exhibited negligible disappearance in the treatment of LCA, LCA/LDB, and LCA/LGG. ANIT‐induced hepatic fibrosis was obviously restored by LCA/LGG‐LDB as the decreased expression of collagenous fibers was presented in LCA/LGG‐LDB treatment group (Figure 2G,I). To further investigate the liver injury alleviation by treating with LCA/LGG‐LDB, the infiltration of inflammatory macrophage was characterized by immunohistochemical labeling of F4/80 marker. After orally administrating ANIT, the high accumulation of inflammatory macrophage in liver parenchyma verified that ANIT has caused serious liver inflammation, and the inflammatory response was not inhibited by cotreating with LCA, LCA/LDB, and LCA/LGG. But cotreating with LCA/LGG‐LDB, the accumulation of inflammatory macrophage was clearly reduced (Figure 2H). The hepatic inflammatory factors including tumor necrosis factor‐*α* (TNF‐*α*), interleukin‐1*β* (IL‐1*β*), and interleukin‐6 (IL‐6) were also tested for evaluating inflammatory level. As showed in Figure 2J–L, the three inflammatory factors were highly elevated in ANIT treatment group and cotreatment with LCA, LCA/LDB, and LCA/LGG. In LCA/LGG‐LDB treatment, these proinflammatory mediators were significantly lowered. These results proved that LCA/LGG‐LDB can effectively prevent hepatic tissue injury and inhibit the hepatic inflammatory response induced by ANIT administration.

### 2.3 Therapeutic Mechanisms of LCA/LGG‐LDB in ANIT‐Induced Liver Injury

**Figure 3:**
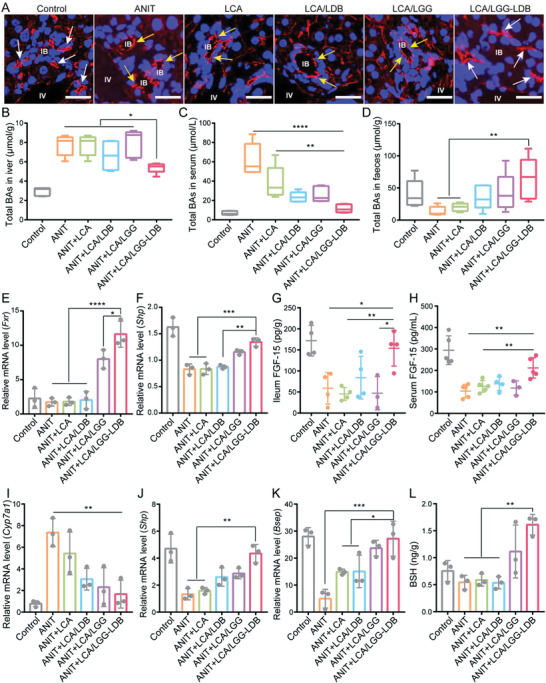
The intervention mechanisms of LCA/LGG‐LDB in ANIT‐induced cholestasis and liver injury. A) Hepatic immunofluorescence staining of ZO‐1 in different treatment groups (White arrows: double rail; Yellow arrows: mono rail; Scale bar: 20 µm). Total BAs in B) liver, C) serum, and D) feces in different treatment groups. Relative mRNA expressions of E) *Fxr*, and F) *Shp* in intestine. The concentration of FGF‐15 in G) ileum and H) liver. Relative mRNA expressions of I) *Cyp7a1*, J) *Shp*, and K) *Bsep* in liver. L) BSH level in feces. The data were presented as the mean ± s.d., *n* = ≈3–5. The statistical significance was calculated via one‐way ANOVA with Tukey's multiple comparisons test. *P < 0.05, **P < 0.01, ***P < 0.001, ****P < 0.0001.

According to previous report, hepatic tight‐junctional structures that seal the bile canaliculus are responsible for providing a barrier to diffusion between bile and blood, which also drive the bile formation by maintaining the bile‐to‐blood gradients.^[^ ^28^ ^]^ Thus, the disorganization of these tight‐junctional complexes can reflect the pathogenesis of cholestasis to some extent. To further clarify the therapeutic effect of the synthesized dual probiotic system in ANIT‐induced liver injury, the integrity of tight‐junctional protein zonula occludens‐1 (ZO‐1) was first analyzed by immunofluorescence staining. As shown in **Figure** **3**A, the location of the train track‐like “double rail” canalicular structures in control group displayed the intact ZO‐1. ANIT treatment induced significant reduction in the number of “double rail” canalicular structures, alternatively, this treatment mainly exhibited spiral‐like “single‐rail” structures, which indicated the serious destruction of the integrity of tight junction structures in hepatic bile canaliculi. This damage was not restored by treatment of LCA, LCA/LDB, and LCA/LGG. After LCA/LGG‐LDB treatment, the “double rail” structures were effectively preserved and the canalicular width was substantially normalized. The destroyed bile duct could cause the permeation of bile into liver tissue and peripheral blood, therefore, BAs were further measured in hepatic tissue and serum. The tested results showed the elevated BAs in both liver tissue and serum by ANIT treatment. The hepatic BAs concentration was not decreased by the treatment of LCA, LCA/LDB, and LCA/LGG, while was significantly lowered by LCA/LGG‐LDB treatment (Figure 3B). In serum, LCA/LGG‐LDB also displayed the significant BAs depuration effect compared to other treatment groups (Figure 3C). These data illustrated that LCA/LGG‐LDB treatment can considerably prevent the hepatic bile duct damage induced by ANIT, and hinder bile to permeate into liver tissue and peripheral blood. Importantly, the elevated BAs were tested in the excreted feces after LCA/LGG‐LDB treatment (Figure 3D), meaning that LCA/LGG‐LDB could promote BAs secretion from liver and intestine.

According to the literature,^[^ ^11^ ^]^ FXR, a member of the steroid/thyroid hormone receptor family of ligand‐activated transcription factors, is associated to BAs synthesis and circulation. Hepatic FXR is involved in the feedback repression of BAs synthesis via small heterodimer partner (SHP) by mainly reducing the expression of a rate‐limiting enzyme of the cytochrome p450 enzymes cholesterol 7*α*‐hydroxylase (CYP7A1) for BAs synthesis. Ileal FXR play critical role in the process of BAs reabsorption through regulating BAs transporter expression. In particular, the activation of ileal FXR can accelerate the expression of a hormone of FGF‐15, which is transported into liver through portal blood to suppress the expression of CYP7A1. Thus, the hepatic FXR and intestinal FXR coordinate to regulate CYP7A1. To research whether the reduction of BAs in liver and serum was also ascribed to activation of FXR‐FGF‐15 signaling pathway, the mRNA expressions of related genes were evaluated. As shown in Figure 3E,F, the mRNA expression levels of ileal *Fxr* and its target gene *Shp* were reduced in ANIT treatment group as well as LCA, LCA/LDB treatment groups, while the mRNA expressions of the two genes were significantly enhanced in LCA/LGG‐LDB treatment group. The concentration of FGF‐15 protein in ileum and serum was further tested. Results showed that the prominent FGF‐15 reduction was found in the groups of ANIT and cotreating with LCA, LCA/LDB, and LCA/LGG. After LCA/LGG‐LDB treatment, the concentration of FGF‐15 was significantly elevated in both ileum and serum (Figure 3G,H). These data demonstrated that the intestinal FXR was activated by LCA/LGG‐LDB treatment. Then, the hepatic mRNA expression of *Cyp7a1* was tested. The tested results revealed that ANIT treatment significantly increased *Cyp7a1* mRNA level, indicating the accelerated BAs synthesis in liver tissue. The high mRNA level of *Cyp7a1* was significantly restored by co‐treating with LCA/LGG‐LDB, suggesting that LCA/LGG‐LDB can decrease hepatic BAs synthesis (Figure 3I). These results were consistent with BAs measurement as shown in Figure 3B,C. The expression of hepatic *Shp* mRNA level was opposite to *Cyp7a1* mRNA level in the same treatment (Figure 3J), which again supported that LCA/LGG‐LDB treatment inhibited CYP7A1 expression and hepatic BAs synthesis. Collectively, these data demonstrated that the hepatic BAs accumulation caused by ANIT can be significantly remitted by LCA/LGG‐LDB cotreatment through activating gut‐liver axis FXR‐FGF‐15 signaling pathway. In order to further explain the high BAs content in feces found in LCA/LGG‐LDB treatment, the mRNA expression of bile salt export pump (*Bsep*), a hepatic BAs transporter, was further detected. After ANIT treatment, the *Bsep* mRNA level was low. But *Bsep* mRNA expression was significantly upregulated after LCA/LGG‐LDB treatment, which indicated that the accumulative BAs in liver could be rapidly excreted into intestinal tract, and finally excreted out along with feces (Figure 3K). The fecal BAs content is also positive correlation with the intestinal bacterial BSH activity as BSH can catabolize hydrophilic conjugated BAs into hydrophobic deconjugated BAs that are more easily excreted out from intestinal tract. Therefore, the fecal BSH was further detected. Results showed that BSH was decreased by ANIT treatment, while treated with LCA/LGG‐LDB, BSH was prominently enhanced (Figure 3L). The data confirmed that the high fecal BAs content in LCA/LGG‐LDB treatment was also attributed to high BSH activity of gut commensal bacteria.

It is worth noting that even if less BSH was presented in LCA/LDB treatment, the obviously increased BAs excretion was detected, which could be ascribed to the absorption and fixation effect of LCA/LDB. Additionally, in comparison with ANIT and LCA/LDB groups, it was found that the intestinal FXR and SHP, hepatic SHP and BSEP, fecal BSH and BAs were obviously elevated in LCA/LGG treatment group, however, the hepatic CYP7A1 was obviously downregulated. These results explained that LGG play crucial role in dredging cholestasis via gut‐liver axis modulation. According to above evidences, we demonstrated that LCA/LGG‐LDB can effectively ameliorate ANIT‐induced cholestasis and liver injury by activating FXR‐FGF‐15 signaling pathway, mediating BAs fixation and guiding deconjugation of intestinal conjugated BAs.

### 2.4 Modulation of Gut Microbiota and Hepatic BAs by LCA/LGG‐LDB

**Figure 4:**
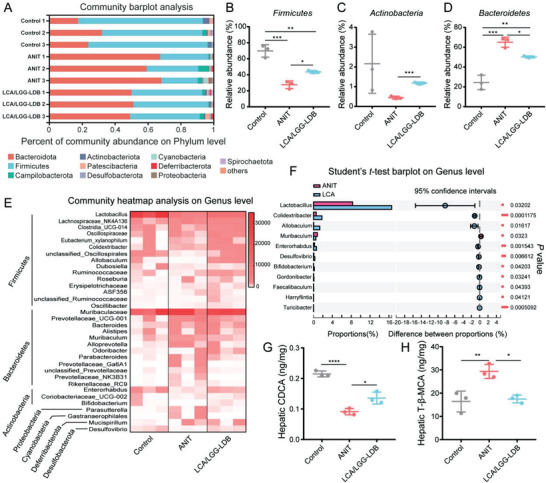
Modulation of gut microbiota and hepatic BAs by LCA/LGG‐LDB. A) Gut microbiota community barplot analysis on phylum level. Relative abundance of B) *Firmicutes*, C) *Actinobacteria*, and D) *Bacteroidetes*. E) Gut microbiota community heatmap analysis on genus level. F) Significance analysis of gut microbiota in genus level between ANIT and LCA/LGG‐LDB treatments. The levels of hepatic G) CDCA and H) T‐*β*‐MCA. The data were presented as the mean ± s.d., *n* = 3. The statistical significance of bacterial genus level was calculated by two‐tailed unpaired student's *t*‐test method. The statistical significance of CDCA and *β*‐MCA were calculated via one‐way ANOVA with Tukey's multiple comparisons test. *P < 0.05, **P < 0.01, ***P < 0.001, ****P < 0.0001.

As shown in Figure 3L, fecal BSH in LCA/LGG‐LDB group presented obvious growth compared to ANIT group, therefore we speculated that BSH expression‐associated commensal bacteria were possibly altered after LCA/LGG‐LDB treatment. Fecal 16S ribosomal RNA gene sequencing was performed for analyzing the compositions change of gut microbiota. Analysis results revealed that bacterial abundance (observed operational taxonomic unit (OTU), Ace index) and the bacterial diversity (Shannon index) not obviously changed after LCA/LGG‐LDB treatment as compared with ANIT and control groups (Figure S8, Supporting Information). The community barplot analysis in phylum level found that Firmicutes and Actinobacteria, known phyla that harbor bacteria with high BSH activity, were highly abundant in control group. After ANIT treatment, the relative abundance of Firmicutes and Actinobacteria were decreased, but Bacteroidetes, a major bacterial phylum that harbor bacteria with low BSH activity was significantly enhanced.^[^ ^29^ ^]^ After LCA/LGG‐LDB treatment, the alteration was restored. The relative abundance of Firmicutes and Actinobacteria were enhanced, and Bacteroidetes was decreased (**Figure** **4**A–D). Further community heatmap analysis on genus level revealed that LCA/LGG‐LDB treatment significantly increased the relative abundance of *Lactobacillus*, *Colidextribacter*, *Allobaculum* of Firmicutes, and *Enterorhabdus*, *Bifidobacterium*, *Gordonibacter* of Actinobacteria, and significantly decreased the relative abundance of *Muribaculum* of Bacteroidetes in comparison to ANIT group (Figure 4E,F). Besides, the relative abundance of *Desulfovibrio* (Desulfobacterota), which is proved to active hepatic FXR and inhibit CYP7A1 expression,^[^ ^30^ ^]^ was also significantly elevated in LCA/LGG‐LDB treatment. The gut microbiota analysis proved that LCA/LGG‐LDB can effectively correct disordered gut microbiota caused by ANIT. The abundance enhancement of highly BSH‐active bacteria induced by LCA/LGG‐LDB indicated the higher intestinal BSH content and more BAs excretion, which was consistent with above BAs and BSH tests. These evidences demonstrated that cholestatic DILI caused by ANIT can be prevented by LCA/LGG‐LDB through modulating the composition and metabolism of gut microbiota.

As mentioned above that the increased total BAs in liver was significantly restored by LCA/LGG‐LDB treatment. To deeply clarify this change, the detailed compositions of hepatic BAs were also tested by targeted metabolomics (Figure S9, Supporting Information). The tested results showed that an FXR agonist of hepatic chenodeoxycholic acid (CDCA) levels were markedly decreased, but an FXR antagonist of taurine‐*β*‐muricholic acid (T‐*β*‐MCA) concentration was significantly elevated in ANIT‐treated mice. The two BAs levels were reversed by LCA/LGG‐LDB treatment (Figure 4G,H). These results verified that LCA/LGG‐LDB treatment may facilitate hepatic FXR activity, reduce CYP7A1 expression through altering hepatic BAs compositions, finally leading to inhibition of BAs synthesis and reducing total BAs in liver.

### 2.5 Remission of VPA‐Induced Hepatotoxicity by LCA/LGG‐LDB

**Figure 5:**
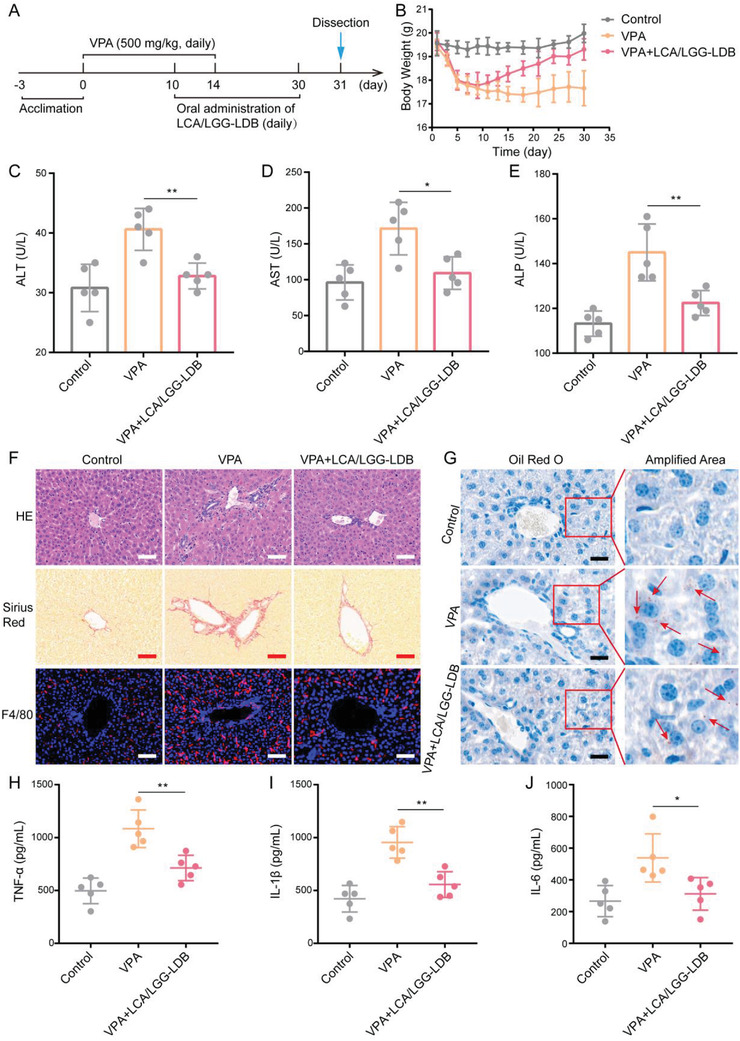
Remission of VPA‐induced liver hepatotoxicity by LCA/LGG‐LDB. A) The therapeutic process of VPA‐induced hepatotoxicity using LCA/LGG‐LDB. B) Mouse body weight changes during treatment. The concentration of liver functions‐related enzymes in serum including C) ATL, D) AST, and E) ALP. F) Hepatic pathological section of H&E staining, Sirius Red staining and F4/80 marker immunofluorescence staining, respectively (Scale bar: 50 µm). G) Oil red O staining of mouse liver tissues (Scale bar: 20 µm). The concentration of hepatic inflammatory factors including H) TNF‐*α*, I) IL‐1*β*, and J) IL‐6, respectively. The data were presented as the mean ± s.d., *n* = 5. The statistical significance was calculated via one‐way ANOVA with Tukey's multiple comparisons test. *P < 0.05, **P < 0.01.

Many clinical drugs have been confirmed to enable to cause drug‐induced cholestasis, which could elicit serious hepatic complications.^[^ ^31^ ^]^ Particularly, due to the demand of long‐term use of medication for suppressing the seizures in epileptic patients, the antiepileptic drugs like VPA, gabapentin, and lamotrigine have been manifested to elicit cholestasis with a high probability.^[^ ^32^, ^33^ ^]^ Next, the therapeutic effect of the synthesized dual probiotics system toward high dose of VPA‐induced cholestasis and corresponding liver injury was evaluated. The therapeutic process with a strategy of halfway drugs administration to imitate real cholestasis occurrence and management in epileptic patients was shown in **Figure** **5**A. LCA/LGG‐LDB began to be administrated in day 10, while VPA treatment was finished in day 14. Totally, a 20‐day course of treatment using LCA/LGG‐LDB was performed. During therapeutic process, an obvious loss of mouse body weight was found after VPA treatment, but the body weight loss can be rapidly recovered by LCA/LGG‐LDB treatment (Figure 5B). The liver functions‐related enzymes including ALT, AST, and ALP presented a substantial growth after VPA treatment, which was inhibited by following LCA/LGG‐LDB treatment (Figure 5C–E). Bilirubin levels including total bilirubin and direct bilirubin also showed significant increase after VPA treatment, which were decreased by LCA/LGG‐LDB treatment (Figure S10, Supporting Information). Mouse hepatic pathological sections were analyzed for further evaluating the therapeutic effect of LCA/LGG‐LDB in VPA‐induced liver injury (Figure 5F). In VPA treatment group, H&E staining exhibited serious cell necrosis in liver parenchyma, portal edema, and diffuse bile duct hyperplasia, while these lesions were normalized by LCA/LGG‐LDB treatment. Sirius red staining revealed the elevated collagen deposition and increased portal fibrosis in VPA‐treated group, but undergoing LCA/LGG‐LDB treatment, the fibrosis of mouse liver was visibly relieved. Additionally, oil red O staining also manifested that VPA treatment can cause hepatic tissue steatosis as the fat granules (red arrows) diffused throughout the liver parenchyma as shown in Figure 5G. The liver steatosis was significantly remitted by LCA/LGG‐LDB as the few fat granules were observed after LCA/LGG‐LDB treatment. The hepatic inflammation was evaluated by labeling inflammatory macrophage marker of F4/80 via immunofluorescence staining. Results showed that VPA treatment caused a massive infiltration of macrophages, demonstrating the serious inflammatory response (Figure 5F). The elevation of inflammatory factors including TNF‐*α*, IL‐1*β*, and IL‐6 also agreed with the severe inflammation in liver tissue caused by VPA (Figure 5H–J). Nevertheless, the evoked inflammation by VPA can been greatly ameliorated after LCA/LGG‐LDB treatment as the macrophages were rarely recruited and the inflammatory factors were significantly reduced (Figure 5F–J). These evidences testified that LCA/LGG‐LDB can markedly prevent VPA‐induced hepatotoxicity.

### 2.6 Prevention Mechanisms of VPA‐Induced Liver Injury by LCA/LGG‐LDB

**Figure 6:**
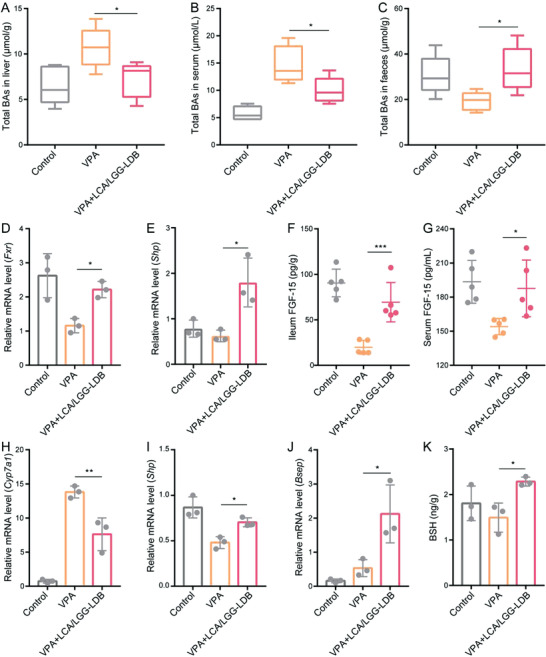
The mechanism of LCA/LGG‐LDB mediated hepatoxicity remission in VPA‐induced cholestasis. Total BAs in A) liver, B) serum, and C) feces. Relative mRNA level of D) *Fxr* and E) *Shp* in ileum. The concentration of FGF‐15 in F) ileum and G) serum. Relative mRNA level of H) *Cyp7a1*, I) *Shp*, and J) *Bsep* in liver. K) BSH content in feces. The data were presented as the mean ± s.d., *n* = ≈3–5. The statistical significance was calculated via one‐way ANOVA with Tukey's multiple comparisons test. *P < 0.05, **P < 0.01, ***P < 0.001.

To evaluate the prevention mechanisms of LCA/LGG‐LDB toward VPA‐induced cholestasis and corresponding hepatotoxicity, total BAs in liver and serum were first tested. Results showed that hepatic and serosanguineous BAs were obviously increased after VPA treatment, while the elevation was abrogated after LCA/LGG‐LDB treatment (**Figure** **6**A,B). Fecal BAs were decreased after VPA treatment, which was ascribed to VPA‐induced hepatic cholestasis. When supplemented with LCA/LGG‐LDB, fecal BAs were significantly elevated (Figure 6C). These data indicated that LCA/LGG‐LDB could scavenge BAs in VPA‐induced cholestasis. The mRNA expression of intestinal *Fxr* as well as its target gene *Shp* were further assessed by polymerase chain reaction (PCR) method. It was found that mRNA expressions of both *Fxr* and *Shp* were lowered after VPA treatment, which were upregulated by LCA/LGG‐LDB treatment (Figure 6D,E). Similarly, the concentration of FGF‐15 in ileum and serum were also decreased after VPA treatment. When treated with LCA/LGG‐LDB, FGF‐15 in both ileum and serum was significantly elevated, meaning that LCA/LGG‐LDB treatment inhibited hepatic BAs synthesis through activating FXR‐FGF‐15 signaling pathway (Figure 6F,G). To verify this speculation, rate‐limiting enzyme of BAs synthesis‐related gene *Cyp7a1* was quantified. Results proved that the upregulated mRNA expression of *Cyp7a1* by VPA treatment was downregulated after LCA/LGG‐LDB treatment, which agreed with the inhibition of hepatic BAs synthesis induced by LCA/LGG‐LDB (Figure 6H). Additionally, mRNA expression of hepatic *Shp* and *Bsep* in LCA/LGG‐LDB treatment were also higher than VPA‐treated group, which revealed the decreased synthesis and accelerated excretion of hepatic BAs (Figure 6I,J). The higher expression of *Bsep* in VPA‐treated group than control group could be ascribed to the activated adaptive mechanism of liver tissue in attempt to attenuate bile acid accumulation and protect against further liver injury.^[^ ^32^ ^]^ Importantly, fecal BSH content in LCA/LGG‐LDB treatment was also higher in comparison to VPA‐treated group, meaning that more conjugated BAs were translated into hydrophobic deconjugated BAs after LCA/LGG‐LDB treatment, which could greatly promote BAs excretion from intestinal tract (Figure 6K). Collectively, these data proved that LCA/LGG‐LDB can effectively ameliorate VPA‐induced cholestasis and liver injury through inhibiting hepatic BAs synthesis and promoting BAs excretion from liver and intestine.

## 3 Conclusions

In this work, probiotic LDB and LGG were hierarchically encapsulated into Ca^2+^‐coordinated polymer microspheres to construct dual probiotics system. The established dual probiotic system exhibited high stability and strictly protected bacterial activity owing to the presence of enteric polymer coating. Charge reversal induced by pH‐dependent dissolution of enteric polymer coating conferred the strong bio‐adhesive ability of the exposed chitosan‐modified calcium alginate microspheres, which tremendously prolonged the retention time of calcium alginate microspheres in intestinal tract and greatly promoted BAs fixation by imprisoned LDB through preadsorbing BAs onto surface of calcium alginate microspheres. In ANIT‐induced mouse model of cholestatic DILI, the dual probiotics system significantly activated FXR‐FGF‐15 signaling pathway, altered hepatic BAs composition and enhanced BESP expression, which resulted in repressive hepatic BAs synthesis and accelerated hepatic BAs excretion. Meanwhile, the dual probiotics system also changed the composition of gut microbiota, elevated the richness of BSH‐active intestinal commensal bacteria, which are beneficial to BAs excretion from intestinal tract through converting conjugated BAs to deconjugated BAs. Along with BAs fixation effect of LDB‐imprisoned calcium alginate microspheres, the satisfactory scavenging effect of intestinal BAs was eventually achieved. In view of the potent BAs depuration effect of dual probiotics system, ANIT and VPA‐induced cholestasis was signally ameliorated, and corresponding liver injury was healed. This strategy exhibited great potential to serve as a universal strategy for cholestatic DILI intervention.

## Conflict of Interest

The authors declare no conflict of interest.

## Supporting information

Supporting Information

## Data Availability

The data that support the findings of this study are available from the corresponding author upon reasonable request.
