# Supplementary material for: Hierarchy‐Assembled Dual Probiotics System Ameliorates Cholestatic Drug‐Induced Liver Injury via Gut‐Liver Axis Modulation
Source: Adv Sci (Weinh). 2022 Apr 17;9(17):2200986. doi: 10.1002/advs.202200986 (PMC9189648; doi:10.1002/advs.202200986)
Supplement: Supplementary file 1 — Supporting Information [file ADVS-9-2200986-s001.pdf]

ADVANCED SCIENCE
Open Access

Supporting Information

for *Adv. Sci.*, DOI 10.1002/advs.202200986

Hierarchy-Assembled Dual Probiotics System Ameliorates Cholestatic Drug-Induced Liver Injury via Gut-Liver Axis Modulation

*Qi-Wen Chen, Qian-Ru Li, Meng-Wei Cao, Jian-Hua Yan and Xian-Zheng Zhang**

Supporting Information of

Hierarchy-Assembled Dual Probiotics System Ameliorates Cholestatic Drug-Induced Liver Injury *via* Gut-Liver Axis Modulation

Qi-Wen Chen[§]*, Qian-Ru Li*[§]*, Meng-Wei Cao, Jian-Hua Yan and Xian-Zheng Zhang**

Q. -W. Chen, Q. -R. Li, M. -W. Cao, J. -H. Yan and Prof. X. -Z. Zhang

Key Laboratory of Biomedical Polymers of Ministry of Education & Department of Chemistry, Wuhan University, Wuhan 430072, P. R. China

*E-mail: xz-zhang@whu.edu.cn

[§]Q. -W. Chen and Q. -R. Li contributed equally to this paper

Materials. Soybean oil, α-naphthyl isothiocyanate (ANIT) and pepsin were purchased from Aladdin. Sodium alginate, calcium chloride anhydrous, NaOH, HCl, Tween 80, dimethyl sulfoxide (DMSO), KH_2PO_4 and NaCl were obtained from Sinopharm Chemical Regent Co., Ltd. Chitosan (degree of deacetylation > 95%), genipin and fluorescein isothiocyanate (FITC) were supplied by Saen Chemical Technology Co., Ltd. Eudragit® L100-55 was purchased from Evonik Nutrition Care GmbH (Germany). Sodium valproate (VPA) was obtained from Tokyo Chemical Industry Co., Ltd. Trypsin and lysozyme were purchased from Macklin Biochemical Co., Ltd. Cyanine 5-NHS ester and Cyanine-5.5 NHS ester were obtained from Yuanye Biological Technology. 7-Hydroxycoumarin-3-carboxylic acid N-succinimidyl ester was supplied by TCI (Shanghai) Chemical Industry Development Co., Ltd. Sodium cholate (98%) was purchased from Beijing Bailingwei Technology Co., Ltd. MRS broth culture medium was obtained from HuanKai Microbial. Agar was supplied by Beyotime. All the chemicals and bio-chemicals were used without further purification.

Instrumentations. Scanning electron microscope (SEM) image was obtained by using scanning electron microscope (Zeiss Sigma). Blood biochemistry analysis was performed by biochemical auto analyzer (MNCHIP, Tianjin, China). *In vivo* imaging was carried out by Spectrum Pre-clinical *In Vivo* Imaging System (PekinEmer). Fluorescent images were obtained by fluorescence inverted microscope (Olympus IX73P2F). The UV-vis absorbance was measured by UV-vis spectroscopy (Lambda Bio40). The bacterial density was measured using microplate reader (Bio-Rad, Model 550, USA). The zeta potential was measured by a zeta sizer (Nano ZS, Malvern Instruments).

Bacterial culture. *Lactobacillus delbrueckii* subsp. *bulgaricus* (ATCC 11842, LDB) and *Lactobacillus rhamnosus* GG (ATCC 53103, LGG) were purchased from Guangdong Microbial Cultural Collection Center (GDMCC). LDB and LGG were cultured anaerobically in MRS broth at 37 ^{o}C.

Fluorescent labeling of LDB, LGG and chitosan. 8 mL LDB in MRS broth culture medium

was centrifuged (6000 rpm, 3 min), followed by resuspending in 8 mL PBS. Then, 7-Hydroxycoumarin-3-carboxylic acid N-succinimidyl ester (5 mg/mL, 10 μL, dissolved in DMSO) was added to above solution. After an hour of stirring in dark at room temperature, the reactant was centrifuged, and precipitate was washed with PBS until no fluorescence in supernatant. LGG was labelled with Cy5-NHS (1 mg/mL, 10 μL, dissolved in DMSO) in the same way.

1 g chitosan was dissolved in 100 mL of 1% acetic acid solution (w/v), then 100 μL of FITC (0.1 mg/mL, dissolved in DMSO) was added. After an hour of stirring in dark at room temperature, the reactant was dialyzed, follow by freeze drying. The lyophilized powders of FITC-labeled chitosan were redispersed in 100 mL of 1% acetic acid solution (w/v).

Synthesis of hierarchy-assembled dual probiotics system. The synthesis of hierarchy-assembled dual probiotic system (LCA/LGG-LDB) was divided into three steps. Firstly, LDB-encapsulated calcium alginate microspheres were prepared by microemulsion method. Briefly, $CaCl_2$ solution (0.5 M, 5 mL) was added to 40 mL soybean oil dropwise under vigorous stirring at room temperature. LDB solution (OD_{600} = 1.5, 5 mL) was mixed with 5 mL of 1% sodium alginate (w/v), and then was added into 40 mL soybean oil drop by drop under vigorous stirring. Then, sodium alginate-contained soybean oil was added to the $CaCl_2$-contained soybean oil quickly. After one hour of reaction at room temperature, a few drops of Tween 80 were added, followed by centrifugation (3000 rpm, 2 min). The supernatant was discarded and these microspheres were washed by $CaCl_2$ solution.

Next, the resulting microspheres were dispersed in 10 mL of 1% chitosan solution (w/v), followed by one hour of stirring at room temperature. Then, the chitosan-modified microspheres were washed by phosphate-buffered saline (PBS) twice, and then were crosslinked with genipin (10 mM, 10 mL) for 2 h.

Lastly, these microspheres were dispersed into 10 mL $CaCl_2$ (0.5 M) solution, followed by mixing with LGG solution (OD_{600} = 1.0, 5 mL). Injection pump was used to inject 10 mL

L100-55 solution (8 mg/mL) into the mixture at the flow rate of 20 mL/h for 30 min under gentle stirring condition. Then, HCl solution (pH = 1) was added to the mixture to maintain pH = 4.5. After centrifugation (800 rpm, 1 min), microspheres were collected, and then stored in 12.5 mL HCl solution (pH = 4.5) at 4 ^{o}C for use. LCA/LDB, LCA/LGG and LCA were prepared in the same way.

Survival of bacteria in SGF, SIF and BA. 1 mL LCA/LGG solution was added to 9 mL PBS (pH = 7.4), SGF (pH = 3.0, 3.2 g/L pepsin and 2 g/L NaCl), SIF (pH = 6.8, 10 g/L trypsin and 6.8 g/L KH_2PO_4), and sodium cholate (0.3 mg/mL), respectively. These mixtures were incubated with a gentle stirring for 100 rpm at 37 ^{o}C for 1.5 h. After a 3-minute-centrifugation, precipitate was washed with PBS, and then resuspended in 5 mL PBS. After proper dilution, 100 μL solution was coated onto MRS agar plates. The bacterial colonies were counted after a 24-hour-incubation anaerobically at 37 ^{o}C.

Adsorption tests of sodium cholate. 1.2 mL of the synthesized CSA-LDB, CSA microspheres or LDB (OD_{600} = 1.5) stored in 5 mL PBS were mixed with 12 mL sodium cholate solution (3 mg/L), then were stirred gently at 37 ^{o}C. After centrifugation, the supernatant was collected and filtered with a 0.22-μm membrane. Then, 1 mL of the filtered supernatant was added into a 12 mL glass tube and 6 mL of 64% H_2SO_4 was subsequently added. After fully mixing, 1 mL of 0.3% furfural was added and reacted for 30 min at 65 ^{o}C. After cooling to room temperature, the absorbance at 620 nm were measured. In this study, different incubation time points (2 h, 4 h, 8 h and 12 h) were evaluated. The absorption capacity was calculated according to standard curve of sodium cholate solution.

Evaluation of intestinal retention in microfluids device. For the purpose of evaluating intestinal retention, ileum and colon of healthy mice were collected and sticked on the different sides of microfluids device. Cy5.5-labeled LCA/LDB or Cy5.5-labeled LCA/LDB without chitosan modification were incubated with SIF and then circulated at a flow velocity of 0.85 cm/s. The fluorescence intensity was recorded with IVIS at predetermined time points

(0 h, 4 h and 8 h after circulation).

In vivo gastrointestinal retention evaluation. In vivo gastrointestinal retention of LCA/LDB were evaluated with healthy female C57BL/6 mice. Mice were fasted for 12 h before orally administrating Cy5.5-labeled LCA/LDB (300 μL). Then, mice were imaged using IVIS at predetermined time points (0 h, 3 h, 6 h, 9 h and 12 h after administration). LCA/LDB without chitosan modification was used as control group. After IVIS imaging, mice were euthanized, and gastrointestinal tracts were collected and imaged.

ANIT-induced cholestasis and liver injury model. C57BL/6 female mice were randomly divided into six groups: control group, ANIT group, ANIT + LCA group, ANIT + LCA/LDB group, ANIT + LCA/LGG group and ANIT + LCA/LGG-LDB group. Mice in latter five groups were orally administrated with 100 μL of ANIT (12 mg/mL, dissolved in soybean oil, once a day for two days), while mice in control group were administrated with equal volume of soybean oil. Two days later, mice in latter five groups were gavaged with 500 μL of the stored materials once a day for twelve days, while mice in control group were administrated with equal volume of PBS. After a-twelve-days treatment, faeces were collected for further analysis. Then, all mice were euthanized, peripheric blood, liver tissue and ileum were collected for further tests and analysis.

VPA-induced cholestasis and liver injury model. In VPA-induced cholestasis and liver injury model, mice were randomly divided into three groups: control group, VPA-treated group (VPA), as well as LCA/LGG-LDB treated group (VPA+LCA/LGG-LDB). Mice were administrated with 100 μL of VPA (100 mg/mL) *via* oral administration in every morning for two weeks, while the control group were orally administrated with equal volume of PBS. In the tenth day, mice in treatment group were orally gavaged with 500 μL of LCA/LGG-LDB in every night for twenty days. Mice in control group and VPA group were administrated with equal volume of PBS. In the end of this experiment, all mice were sacrificed, and then blood, faeces, liver tissue and ileum were collected for further tests and analysis.

Serum biochemical analysis. Serum was collected by centrifugation from whole blood sample at the speed of 3200 rpm at 4 ^{o}C for 15 min. Serum ALP, ASP, AST, total bilirubin and direct bilirubin were detected by liver and kidney function test lyophilization kit (MNCHIP) according to the manufacturer's protocol.

Determination of BSH in mouse faeces. Briefly, 0.5 mL of lysozyme solution (0.5 mg/mL) was added to a mouse fecal sample, and then mixture was incubated at 37 ^{o}C for 10 minutes. After homogenization, the mixture was centrifuged at 4 ^{o}C, and supernatant was collected. BSH in supernatant was determined by Mouse Bile Salt Hydrolase Elisa Kit (Jiangsu MEIMIAN industry Co., Ltd) according to manufacturer's protocol.

16S rDNA sequencing, Real-time PCR studies, and measurements of total BAs, FGF-15, and inflammatory factors. The high-throughput 16S rDNA sequencing was performed in Majorbio Bio-Pharm Technology Co. Ltd. The data were analyzed on the free online platform of I-Sanger Cloud Platform. The primers of Real-time PCR experiments were adopted according to previous reports[47], and the producers were performed by using standard laboratory assays. Total BAs, FGF-15, and inflammatory factors were measured by using Elisa Kit (Total BAs Elisa Kit was purchased from Nanjing Jiancheng Bioengineering Institute (China); FGF-15 Elisa Kit and inflammatory factors Elisa Kit including TNF-α, IL-1β, IL-6 were obtained from Beijing Solarbio Science & Technology co., Ltd) according to the manufacturer's instructions.

Statistical Analysis. All values ($n \geq 3$) were represented as the mean ± standard deviation (SD). Statistical analysis was performed using GraphPad Prism 8.0 software and two-tailed unpaired Student's *t*-test or one-way analysis of variance (ANOVA) followed by Tukey's multiple comparisons test was used to compare differences between treatment groups. Differences were considered statistically significant at *$P < 0.05$, **$P < 0.01$, ***$P < 0.001$, ****$P < 0.0001$.

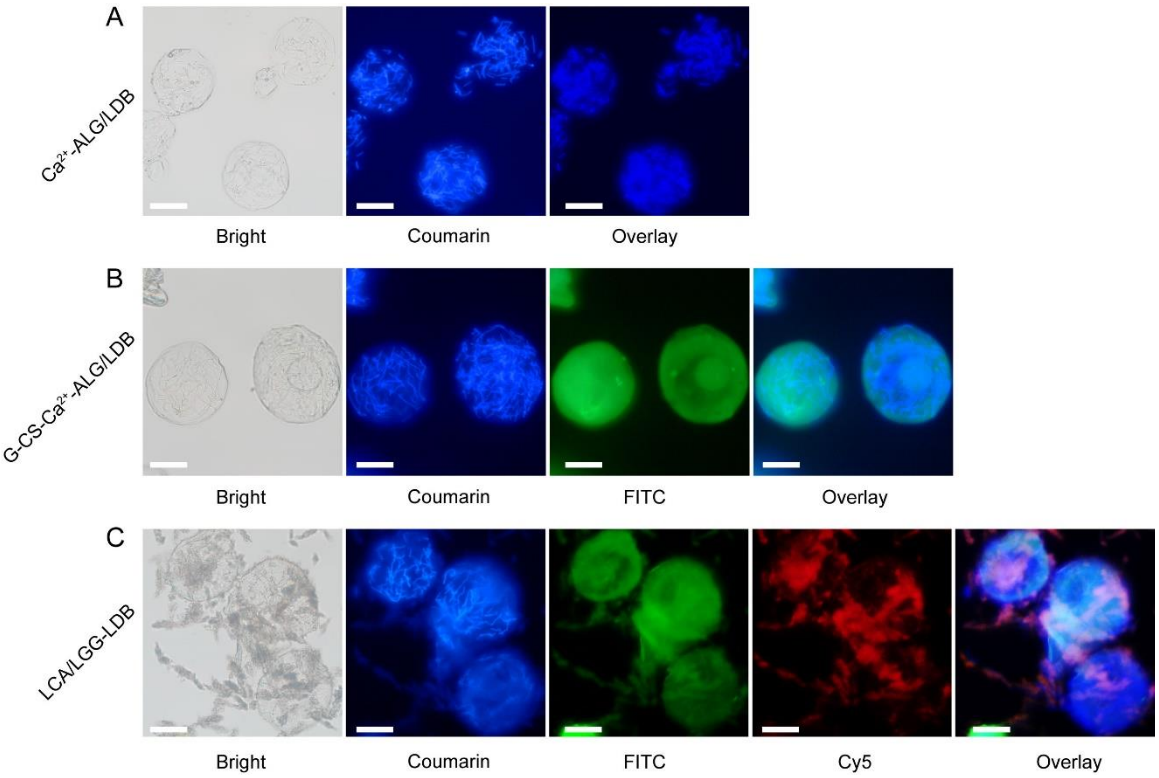

Figure S1. Images of the synthesized materials. (A) Calcium alginate microspheres encapsulating LDB (Blue: coumarin-labeled LDB; Scale bar: 50 μm). (B) Chitosan-modified calcium alginate microspheres (Green: FITC-labeled chitosan; Scale bar: 50 μm). (C) Chitosan-modified calcium alginate microspheres incorporating LGG and coated with enteric polymers (Red: Cy5-labeled LGG; Scale bar: 50 μm).

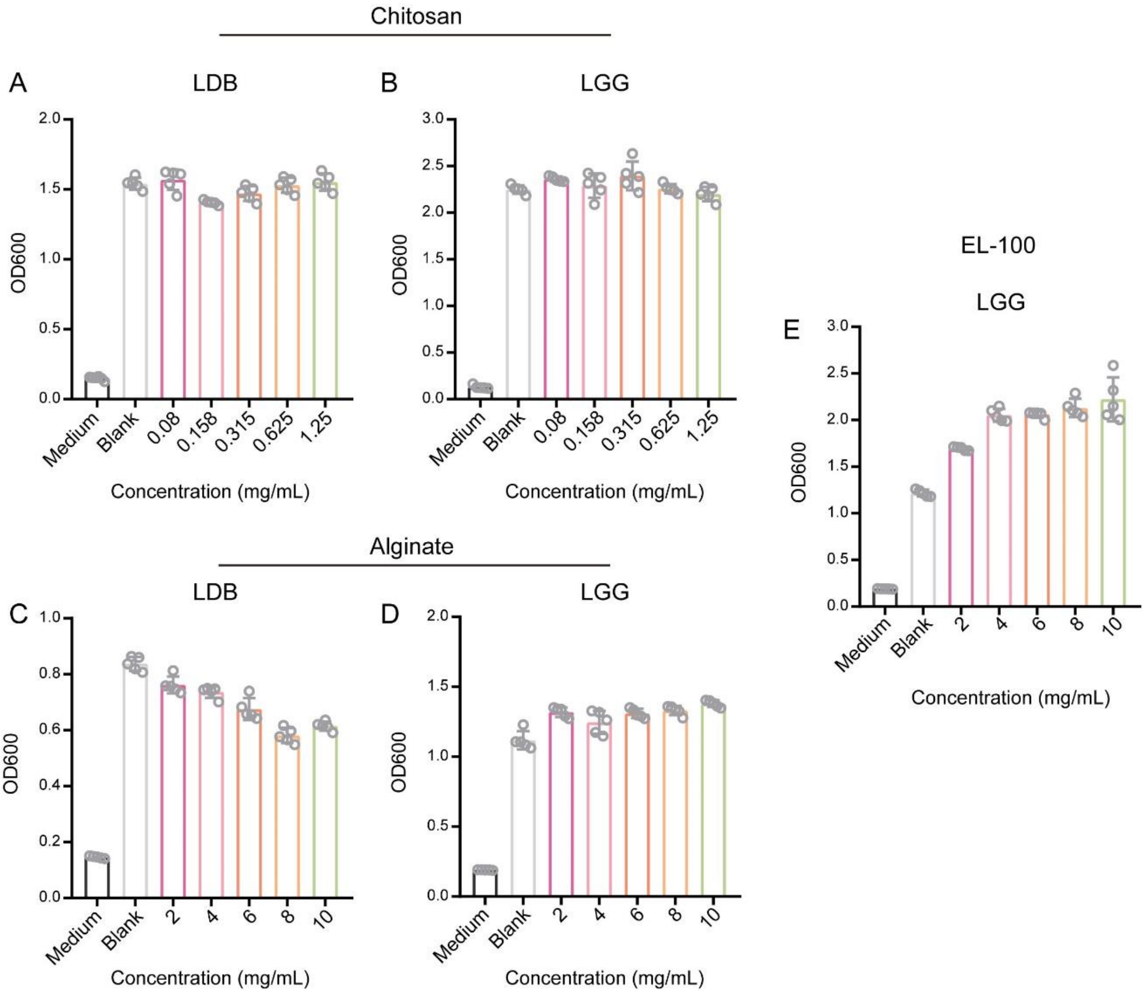

Figure S2. The bacterial cell toxicity of various components toward LDB and LGG. (A, B) The bacterial cell toxicity of chitosan toward LDB and LGG, respectively. (C, D) The bacterial cell toxicity of sodium alginate toward LDB and LGG, respectively. (E) The bacterial cell toxicity of EL-100 toward LDB. n = 5.

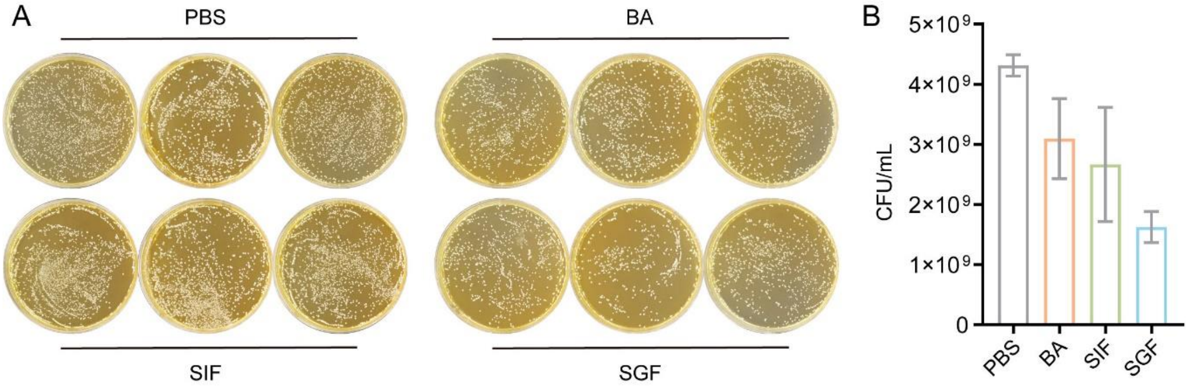

Figure S3. (A) LGG colonies in MRS agar plates after incubating LCA/LGG in different simulated physiological fluids, including PBS (pH = 7.4, 1.5 h), sodium cholate (CA)-added PBS (pH = 7.4, CA: 0.3 mg/mL, 1.5 h), SIF (pH = 6.8, 10 g/L trypsin + 6.8 g/L KH_2PO_4, 1.5 h) and SGF (pH = 3.0, 3.2 g/L pepsin + 2 g/L NaCl, 1.5 h). All were diluted for 10^5 times. (B) The quantification of LGG colonies. n =3.

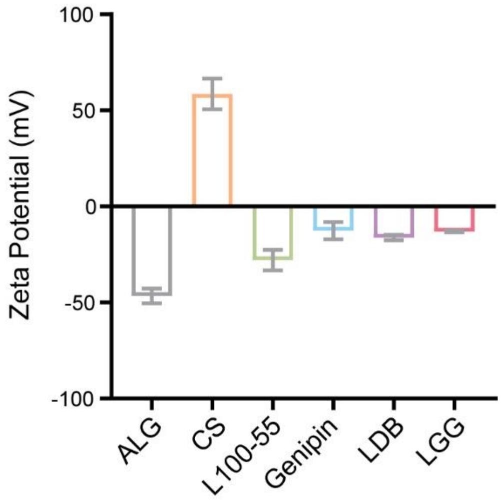

Figure S4. The zeta potential of the used components (including alginate (ALG), chitosan (CS), enteric polymers (L100-55), LDB and LGG). n = 3.

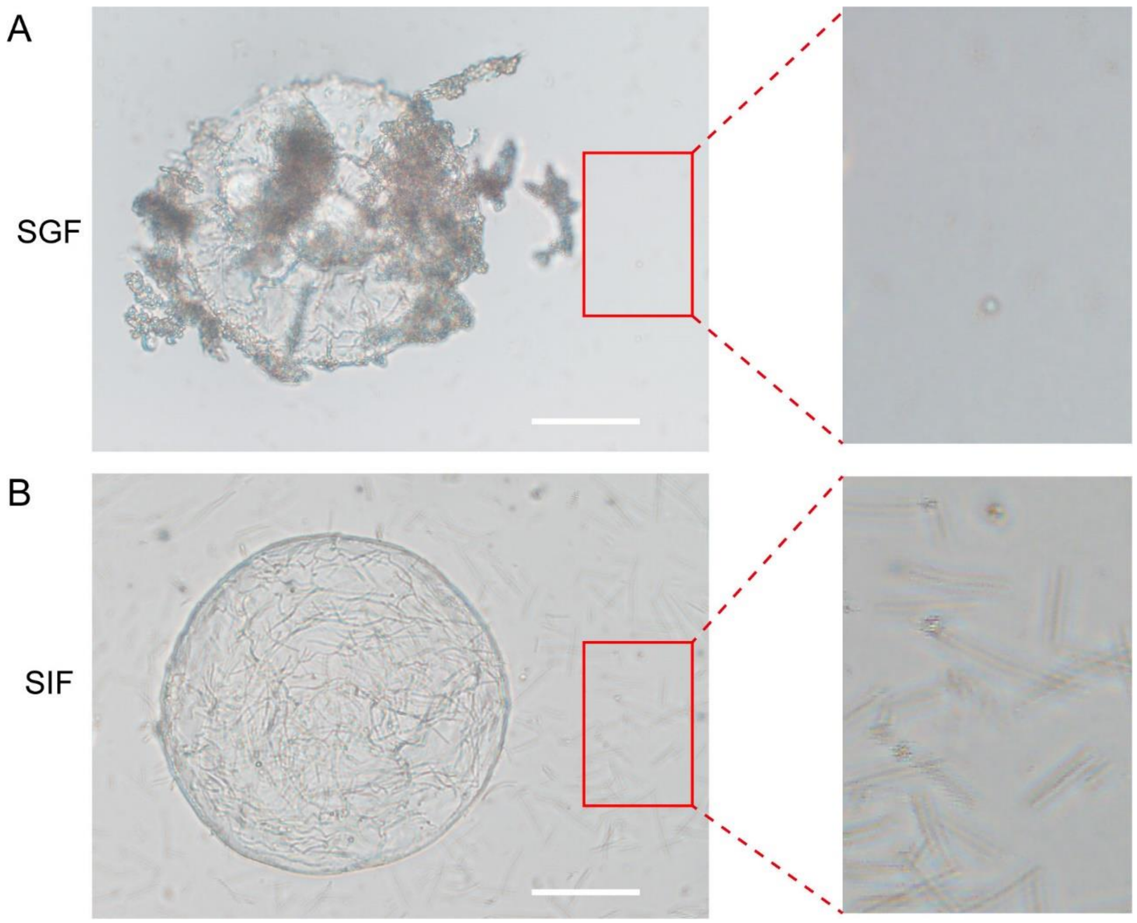

Figure S5. The protection and release of LCA/LGG-LDB in simulated physiological fluids. The degradation behavior of LCA/LGG-LDB in (A) SGF (pH = 3, 1.5 h) and (B) SIF (pH = 6.8, 1.5 h). The enlarged image showed the released LGG in SIF while it was absent in SGF. Scale bar: 50 μm.

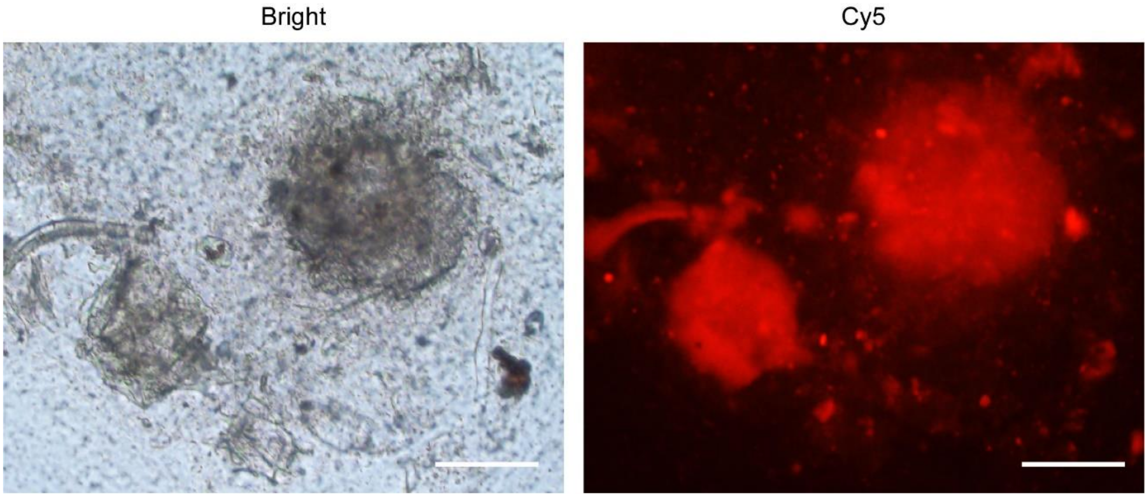

Figure S6. The images of the excreted microspheres in faeces (Scale bar: 100 μm).

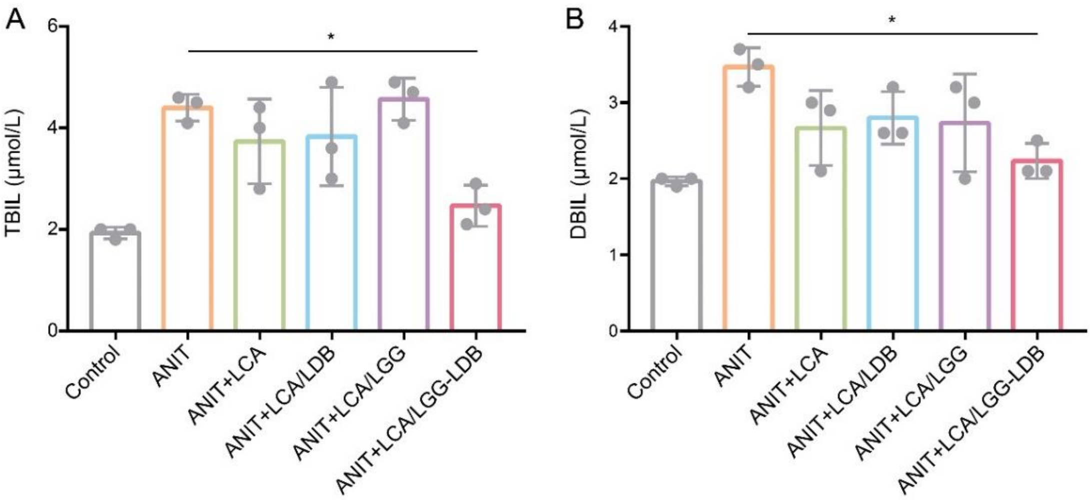

Figure S7. The bilirubin levels in serum (ANIT-induced cholestasis). (A) Total bilirubin. (B) Direct bilirubin. The data were presented as the mean ± s.d., n = 3. The statistical significance was calculated *via* one-way ANOVA with Tukey's multiple comparisons test. **P* < 0.05.

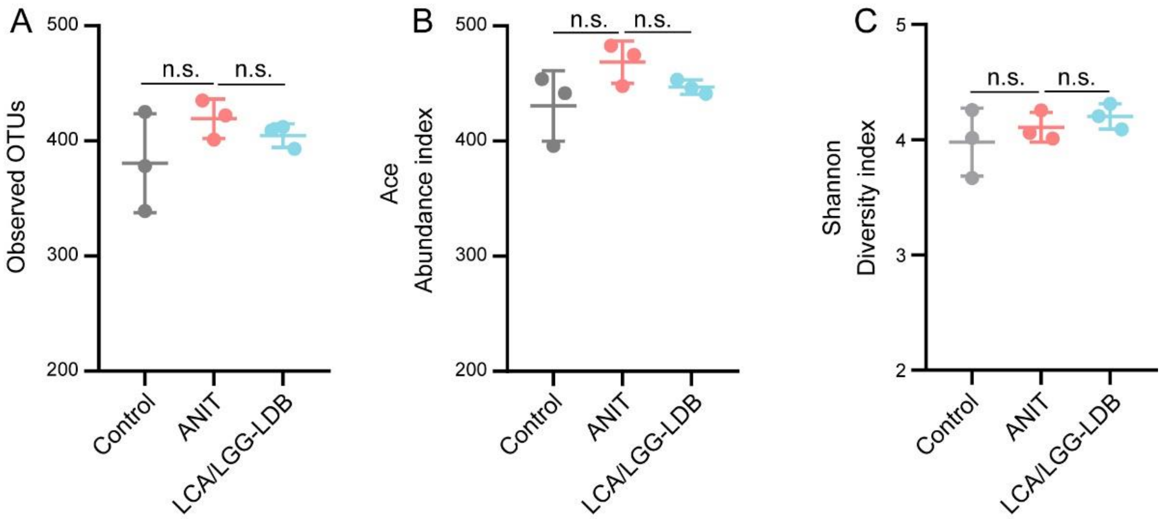

Figure S8. Evaluation of gut microbiota abundance and diversity in the treatment of ANIT and LCA/LGG-LDB. (A) Observed operational taxonomic unit (OTU). (B) Ace index. (C) Shannon index. The data were presented as the mean ± s.d., n = 3. The statistical significance was calculated *via* one-way ANOVA with Tukey's multiple comparisons test (n.s. = no significance).

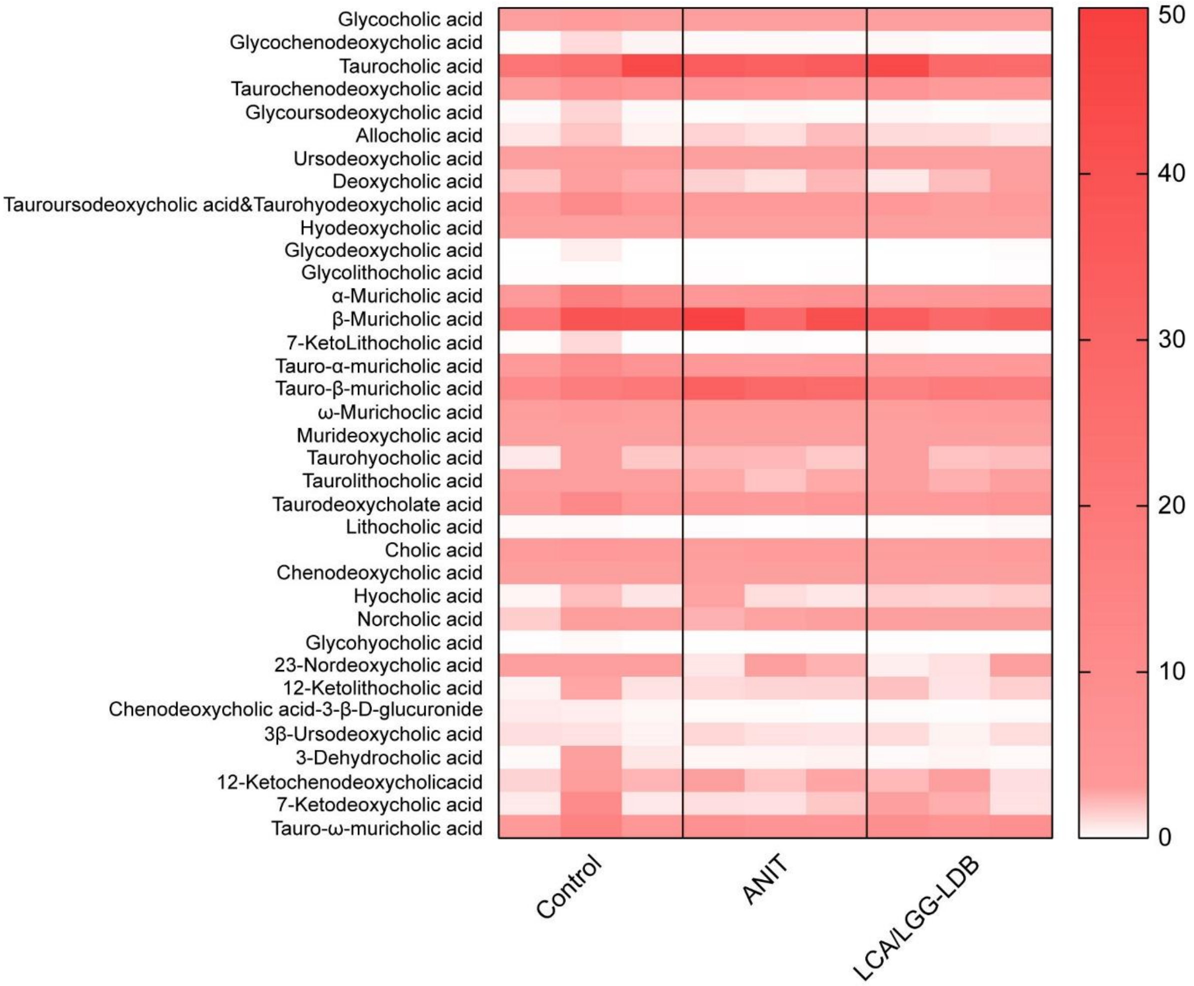

Figure S9. Heat map of hepatic BA compositions.

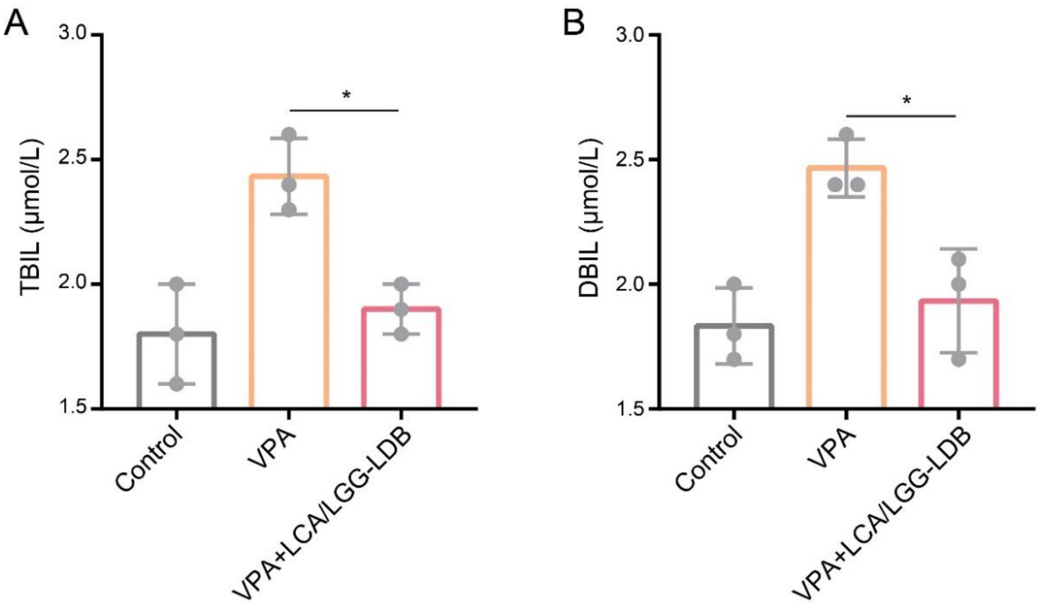

Figure S10. The bilirubin levels in serum (VPA-induced cholestasis). (A) Total bilirubin. (B) Direct bilirubin. The data were presented as the mean ± s.d., n = 3. The statistical significance was calculated *via* one-way ANOVA with Tukey's multiple comparisons test. *$P < 0.05$.
